# The role of multidisciplinary MS care teams in supporting lifestyle behaviour changes to optimise brain health among people living with MS: A qualitative exploration of clinician perspectives

**DOI:** 10.1111/hex.14042

**Published:** 2024-04-04

**Authors:** Olivia Wills, Yasmine Probst, Jodi Haartsen, Anne‐Therese McMahon

**Affiliations:** ^1^ School of Medical, Indigenous and Health Sciences University of Wollongong Wollongong New South Wales Australia; ^2^ MS Plus Ltd. Blackburn Victoria Australia

**Keywords:** behaviour, brain health, healthcare, lifestyle, multiple sclerosis, qualitative research

## Abstract

**Introduction:**

Healthcare professionals have an important role in advocating for the adoption of a brain‐healthy lifestyle for optimal multiple sclerosis (MS) care. Nonetheless, studies to date have mainly focussed on the consumer perspective. Herein, we aimed to explore the current practices of how healthcare professionals support protective, lifestyle‐related behaviour changes to optimise brain health among people living with MS (plwMS), and their perspectives of professional roles.

**Methods:**

Australian healthcare professionals were recruited via study advertisements, purposive and snowball sampling, to participate in an online, semi‐structured and audio‐recorded interview. Clinicians were eligible if they had a minimum of a tertiary Bachelor's degree and 12‐months experience working with plwMS, access to the Internet and sufficient time to participant. An inductive, data‐driven form of reflexive thematic analysis was undertaken before thematic categorisation of the quotes from transcripts. Data analysis was guided by the methods of Braun and Clark and the study's underpinnings drew on the constructs of the Social Cognitive Theory (SCT).

**Results:**

Six physicians, 10 MS nurses, 18 allied health professionals, one exercise therapist and one alternative therapist were interviewed. Three primary themes encompassing the perceived role of healthcare professionals in supporting a brain‐healthy lifestyle were identified: (1) the empowering role, (2) collaborative role and (3) communicative role. External factors/forces including time constraints, professional expertise, training and skill set, power dynamics, consumer readiness, health literacy, self‐efficacy and motivation are at play, and affect how/when healthcare professionals may support behaviour change to optimise lifelong brain health for plwMS.

**Conclusion:**

Healthcare professionals recognise their critical role in encouraging and supporting the adoption of a brain‐healthy lifestyle to optimise lifelong brain health for plwMS. However, discord is evident when they underestimate the complexity of translating knowledge of lifestyle‐related behaviour change(s) into action. Greater awareness must be made in recognising and addressing the bidirectionality of external factors such as those in the SCT, that may influence how behaviour change occurs.

**Public Contribution:**

Healthcare professionals volunteered to be interviewed as part of the data collection phase of this study.

## 1 INTRODUCTION

The number of people living with multiple sclerosis (MS) is rising at a significant and accelerating rate, affecting over 2.9 million people worldwide and over 33,000 people in Australia.^1^ Together with the increasing prevalence and issues related to tolerability, safety, compliance/adherence and efficacy of disease modifying therapies (DMT's) which aim to slow disease progression,^2^, ^3^ nonpharmacological interventions are gaining increased attention for MS care to promote neurological reserve and maximise lifelong brain health.^4^, ^5^ This coincides with an increased need for comprehensive healthcare.

The adoption of a ‘brain‐healthy lifestyle’, which may include physical activity, avoidance of smoking and a healthy diet, are vital to the daily management of MS and have been found to improve the overall quality of life of people living with MS (plwMS).^6^ Further to this, there is growing evidence that positive behaviour changes are associated with improved health outcomes. Specifically, studies have reported that avoiding smoking, limiting alcohol intake, regular physical activity, and a healthy diet in MS, may reduce the risk of disease progression^7^ and rate of relapse,^8^ while also reducing disability.^9^, ^10^ Therefore, given the importance of lifestyle‐related risk factors associated with advancing MS, exacerbation of symptoms and the presence of comorbidities, clinicians need to intervene by addressing consumer lifestyle as a part of person‐centred care.^9^

In the context of MS, clinicians have a responsibility to educate, advocate and provide evidence‐informed advice during routine consultations.^11^ In particular, physicians develop therapeutic relationships with plwMS over time, and are, therefore, considered a trusted source of behaviour change advice.^12^ The clinician's role encompasses raising awareness, motivating their patients/clients and strengthening commitment in those who are not yet considering or are not ready for change.

While clinicians undoubtedly play a pivotal role in supporting plwMS through behaviour change, studies have revealed consumer challenges in obtaining reliable and consistent information regarding lifestyle modifications. PlwMS have also expressed disappointment and dissatisfaction with how lifestyle management, or the lack thereof, was incorporated into their neurology consultations, with a desire for healthy lifestyle management to become central to routine healthcare discussions alongside DMT's.^13^ Limited studies that have explored clinician perspectives have only focussed on MS speciality neurologists and nurses across Australia, and predominantly concentrated on single behavioural change strategies, for example, smoking cessation.^14^ Despite this, the MS care team is inclusive of allied health professionals who also have an important opportunity to support plwMS towards behaviour change.

Allied health professionals have a vital role in the success of many medical interventions and for enhancing and maintaining the wellbeing of their clients.^15^ Multidisciplinary collaboration between physicians, the nursing team and allied health professionals can augment lifestyle changes that are more likely to be sustained in the long term and have been shown to improve the quality of care.^16^ As very little is known about how MS clinicians value and support lifestyle behavioural changes, exploring their perspectives will enhance our understanding of how a brain‐healthy lifestyle is supported in practice, and may enhance consumer engagement efforts to achieve optimal brain health for all plwMS.

Therefore, the primary aim of this study was to qualitatively explore the current practices of how/if healthcare professionals support a brain‐healthy lifestyle/behaviour change among plwMS, and their perspectives of their professional role(s) in this context. The objectives were to:

1. identify how/if clinicians value the concept of brain health and a brain‐healthy lifestyle.
2. describe clinician perceptions of their role(s) in supporting a brain‐healthy lifestyle/behaviour change.
3. explore current practices for the provision of lifestyle‐related behaviour change advice.

## 2 METHODS

### 2.1 Theoretical framework

While the theoretical approach used in this study was an inductive approach using a relativist research epistemology,^17^ the study's underpinnings draw on constructs of Bandura's Social Cognitive Theory (SCT).^18^, ^19^ The SCT posits that personal and environmental factors influencing healthcare professional's practice can have a significant and bidirectional impact on uptake and translation of positive health behaviours.^19^ This theory was chosen to help explain sociostructural and personal determinants that may influence how healthcare professionals support behaviour change, influencing their perspectives of professional roles.^18^ We have also drawn on the work of Swift et al.^20^ and Richard et al.^21^ which guided the design and implementation of collecting and analysing qualitative data. The COnsolidated criteria for REporting Qualitative research^22^ was applied to ensure adherence with reporting of qualitative research (Supplementary File 1). Ethical approval for this study was obtained from the University of Wollongong's Human Research Ethics Committee (reference number: 2022/334).

### 2.2 Participant selection

#### 2.2.1 Recruitment and sampling

Clinicians were recruited from January to July 2023 through study advertisements shared by MS Australia and MS Nurses Australasia, social media (Twitter, LinkedIn, Facebook) accounts of the research team to engage professional online networks and purposive sampling using emails distributed to Australian MS clinics identified on the MS Australia website. Snowballing techniques from participating healthcare professionals were also applied.^23^ Physicians (i.e., neurologists or general practitioners) with a special interest or expertise in MS management, nurses working in the speciality of MS, allied health professionals and, therapists were considered for inclusion if they had a minimum of a tertiary Bachelor's degree and 12‐months experience working with plwMS, access to the Internet and sufficient time to participate. Eligibility was also restricted to those residing and working in Australia at the time of their interview, with the intent to use the findings to inform future guideline development for Australian healthcare professionals working with plwMS. If eligibility was met and informed consent was obtained, clinicians were invited to participate in an online, semi‐structured interview. Participants were provided a $50 voucher for their time.

### 2.3 Data collection

MS clinicians were interviewed via Zoom (Zoom Video Communications Inc., Version: 5.13.6), between 19 April to 1 August 2023. Interviews were conducted virtually to allow for interstate clinicians to participate. Each interview was scheduled for a period of up to 60 min. Interviews were chosen as the most appropriate qualitative data collection method to obtain in‐depth feelings and perspectives related to the research aim and, for practical reasons. That is, it is challenging to schedule focus groups with a number of healthcare professionals as they are often limited by time in their practice. Before the scheduled interview, participants completed a short questionnaire to gather information on demographics including age, gender, profession, education, and clinical experience.

A semi‐structured interview guide (Supplementary File 2, Table S1) was developed in advance of the interviews, using constructs of the SCT, to provide a transparent framework and ensure that key topics were addressed. Interview questions followed the funnel technique^24^ to allow for a unique interpretation of experiences that had significant meaning to the participant. Probing questions were used to expand on individual responses and/or provide example(s) from practice. The interview guide was assessed for face‐validity before use and pilot tested with two senior members of the research team (Y. P., A. T. M.) for review and relevance of the open‐ended questions.

To allow for differences in concept, the moderator first inquired on the participant's view about the meaning of brain health and a brain‐healthy lifestyle in the context of MS. Additional questions related to the clinician's role, current practices, support, multidisciplinary care, and attitudes towards supporting a brain‐healthy lifestyle among plwMS (Table S1).

Memos and field notes were documented during the interview process (E. D. or E. W. M.) which included changes in tone, pauses and gestures that may indicate points of emphasis to assist with data analysis. Interviews were professionally transcribed verbatim using Otter.ai (www.otter.ai.com) and audio verified (O. W., E. D., E. W. M., S. A. I.) by comparing each written transcript to the audio recording and correcting discrepancies. This process of verification in qualitative research enhances quality assurance and validity of the data.^25^ For participant confidentiality, all data were deidentified, pronouns were neutralised using they/their and participants were assigned a code to preserve anonymity. Participants had the opportunity to review their transcript before the commencement of data analysis. No repeated interviews were conducted.

### 2.4 Data analysis

An inductive, data‐driven form of analysis was undertaken, where themes were constructed using complete semantic coding, representing the team's explicit understanding of the data. Reflexive thematic analysis was iterative and followed the six phases proposed by Braun and Clark.^17^ Transcripts were reread for familiarisation and the primary researcher (O. W.) kept reflective notes to consider common patterns. Initial semantic coding was manually conducted line‐by‐line by three independent analysts (O. W., E. D., E. W. M.) to identify simple codes that focused on the explicit meaning of what was said by the participants. The primary analyst (O. W.) developed a single codebook which was reviewed by the research team and refined over time. These codes were foundational to identifying key themes from the data.

Codes were then grouped into like categories of latent meaning to identify candidate themes and subthemes. Themes (defined a priori as a patterned response or meaning with a data set)^17^ were identified based on the analysts perceptions of the broad patterns of meaning across the data set and were iteratively revised and reviewed through discussion between members of the research team (O. W., Y. P., J. H., A. T. M.) to help ensure agreement across findings.^17^ NVivo (QSR International Pty Ltd., v1.7.1) was used to manage the digitally coded data.

The research team concurrently collected and analysed participant interviews until the number of new and/or emerging themes were efficiently and effectively saturated within this group of participants. Strauss et al. previously suggested that a sample size of *n* = 18–20 is adequate to achieve data saturation in exploratory, qualitative research and was, therefore, used as a guide during data collection. Sociodemographic variables were summarised using descriptive statistics in IBM SPSS software (IBM Corp., Released 2021, IBM SPSS Statistics for Windows, Version 28.0; IBM Corp).

### 2.5 Researcher characteristics and reflexivity

The research team comprised of a student dietitian, student exercise physiologist, medical science student, one nurse and three accredited practising dietitians with varying levels of experience in academia and private practice. Members were chosen due to their expertise in MS, nutritional care, and qualitative research, inclusive of relativist research epistemology. To establish rigour and enhance trustworthiness of the data, consideration of the researcher's positionality during the data collection and analysis procedures were critically discussed within the research team. At the time of the interviews, the female moderator and primary analyst (O. W.) was a PhD candidate and had clinical experience working as a dietitian and consulting plwMS. Reflexivity techniques were employed to identify and manage potential researcher bias. By doing so, reflexive diaries were kept by the moderator to reflect on what was being highlighted and silenced during interviews to ensure that the participants continued their own path of personally significant avenues. Critical self‐reflection and peer‐debriefing between the moderator and observer post interview also allowed the moderator to consider how they acted as a contributor in the co‐construction of knowledge and helped to inform and shape future interviews (e.g., by the adjustment of questions or addition of probes). The moderator was previously known to one participant.

## 3 RESULTS

Thirty‐six (36) healthcare professionals met the eligibility criteria, provided informed consent, and were interviewed. The final sample comprised of a heterogenous group of healthcare professionals with varying levels of experience within the MS context. MS speciality nurses (*n* = 10) and dietitians (*n* = 10) had the largest representation, making up 56% of participants. Participants had an average of 10 years (±8.84) consulting experience with plwMS and averaged 12.7 (±13.7) consultations with plwMS per week. Interviews averaged 50 min (±9.8) per interview. No unique codes or themes were identified from the last four interviews and, therefore, recruitment stopped after the 36th participant because of inductive semantic saturation in this context. Participant characteristics are presented in Table 1.

**Table 1:** Participants demographics and characteristics.

Characteristics	No. (%) mean (SD), [min–max]
Age in years	
20–29	5 (14%)
30–39	12 (33%)
40–49	9 (25%)
50–59	6 (17%)
60–69	3 (8%)
70+	1 (3%)
Gender	
Man	6 (17%)
Woman	29 (80%)
Other	1 (3%)
State of residence	
Victoria	13 (36%)
New South Wales	14 (39%)
South Australia	2 (6%)
Queensland	2 (6%)
Western Australia	4 (12%)
Tasmania	0 (0%)
ACT	0 (0%)
New Zealand	1 (3%)
Highest degree or level of education	
Bachelor or Medical degree	12 (33%)
Graduate diploma (incl. master's or graduate certificate)	16 (44%)
Doctorate or higher	8 (22%)
Country of clinical training	
Australia	32 (89%)
UK	2 (6%)
NZ	1 (3%)
US	1 (3%)
Profession	
Alternative therapist	1 (3%)
Dietitian	10 (28%)
Exercise therapist	1 (3%)
MS nurse	10 (28%)
Physiotherapist	3 (8%)
Physician	6 (17%)
Psychologist	3 (8%)
Occupational therapist	2 (6%)
Work setting	
Public (inpatient/outpatient)	16 (44%)
Community/MS clinic	8 (22%)
Private practice	13 (36%)
Executive role/research	4 (12%)
Years of working (clinical) experience	17 years (±13.21), [1–48]
Years of experience consulting people with MS	10 years (±8.84); [1–33]
Number of people with MS seen per week	12.7 (±13.7); [0–50]

Abbreviations: ACT, Australian Capital Territory; MS, multiple sclerosis; NZ, New Zealand; SD, standard deviation; UK, United Kingdom; US, United States.

Only a few clinicians had a well‐rehearsed and clear definition of brain health when asked. Although most clinicians could describe their personal interpretation of the concept, it was perceived and valued differently between participants and, most notably, across professional disciplines. Further, the depth of interpretation of the concept varied according to the discipline of practice and whether brain health was viewed within a biomedical construct:

I'm thinking pretty pathophysiologically on what's the mechanism of action, are there antioxidants that might be available, are there things that might be able to support neuronal regrowth…. (P13, 20:09, Physician)

Others viewed and defined brain health through a holistic lens, likely reflective of their professional discipline:

I think about ways that people can adapt their life to living better with MS and make modifications to their lifestyle… (P16, 14:35, MS nurse)

MS nurses were the greatest advocates for the use of the concept of brain health as a primary MS target:

I see it almost as another disease modifying therapy it's that important. Brain health is everything in multiple sclerosis because we're talking about something that we can't cure, we don't have a magic bullet for it. Brain health is that part of treatment that keeps everything going as much as we can, until we do get a cure … So, for me, brain health is that unmet need at the moment, it's all we have to try and enhance that as much as we can. (P16, 14:35, MS nurse)

**Figure 1:**
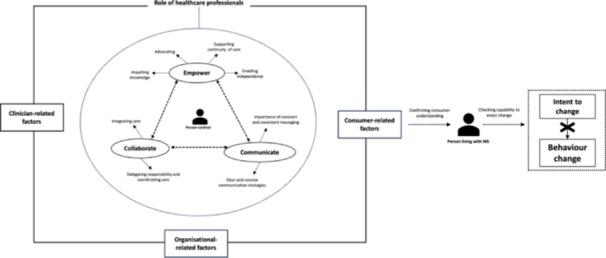
Visual representation of the perceived role of how healthcare professionals support the adoption of a brain‐healthy lifestyle by plwMS. This figure highlights the connections between key themes and external influences/forces shaping how healthcare professionals perceive their role in supporting the adoption of a brain‐healthy lifestyle. External forces with distinct categorisations (i.e., clinician‐related, consumer‐related and organisational‐related factors) are depicted by establishing a firm boundary around key themes/roles identified, denoting the tight boundaries in which healthcare professionals must work. Broken/dotted lines between key themes highlight their dependent and bidirectional relationship. The figure also highlights the shared responsibility between healthcare professionals and plwMS and their readiness to take action. The evident disconnect between confirming consumer understanding and capability to enact change is shown to impede how behaviour change takes action. In this context and as reflected in the results, the intent to act does not always translate to behaviour change. We must note that this figure does not consider the broader social determinants of health and how this may impact on behaviour change as it falls beyond the scope of this research paper. MS, multiple sclerosis.

Inductive thematic analysis of interview transcripts resulted in the identification of 176 semantic codes which were categorised into three overarching themes (Table 2). Here, we present the themes with representative quotations. While discussed separately, many of the themes are interrelated to optimise how clinicians best support lifestyle behaviour change for the adoption of a brain‐healthy lifestyle. This is visually displayed in Figure 1.

**Table 2:** Themes and subthemes identified from the interviews.

Theme (professional role)	Subtheme (i.e., current practices)
1 The empowering role.	1. Imparting knowledge. 2. Varied commitment to brain health advocacy. 3. Enabling independence. 4. Supporting continuity of care.
2 The collaborative role.	1. Integrating care. 2. Delegating responsibility and coordinating care.
3 The communicative role.	1. Clear and concise communication strategies. 2. Importance of constant and consistent messaging.

### 3.1 Theme 1: The empowering role

Healthcare professionals perceived their primary role as **imparting knowledge** to build consumer understanding and preparedness for behaviour change, a foundation to the adoption of a brain‐healthy lifestyle. Participants highlighted the importance of educating plwMS, which relies on translating the evidence from a research base into practical and tailored advice according to the individual needs of the person. By doing so, healthcare professionals considered it an important responsibility of their own to inform consumers on how to best achieve a brain‐healthy lifestyle:

I would say a lot of it at the moment is education of the MS community. So keeping them updated on the benefits of positive health behaviour. And keeping them updated on any outcomes that are available from research that is currently underway elsewhere in the world or within Australian research groups. So, I suppose it's that higher level of one to one but communicating to them the importance of healthy living. (P10, 4:57, Physiotherapist)

Given the complexities of healthcare, provision of education was influenced by several interprofessional, organisational and consumer‐related factors. Across most disciplines, lifestyle management discussions were not given sufficient priority during routine clinical appointments due to time constraints:

So the average consultation is about 15 minutes. That's nationally… That's just a mixture of both patient pressure because you don't get through the patients and people are waiting for years before they can see you… And part of it is financial, because you know, you don't get paid a lot for a consultation. So you got to get through a few consultations in an hour. (P13, 45:46, Physician)

From a consumer perspective, acceptance of healthcare education was also influenced by external factors including one's self‐efficacy, readiness to change, motivation(s) and health literacy. Healthcare professionals rely heavily on consumer readiness to initiate education:

Receiving advice is something that really depends on the client's ability and willingness to get advice. There's no point in me giving information if someone's not here to get information…. (P05, 5:39, Psychologist)

Importantly, healthcare professionals emphasised that the impetus to change is driven largely by involvement and the responsibility of plwMS to act on the information and advice provided:

As long as they're informed. Informed decision making is the most important thing … they just have all the information and then it's up to the patient. (P08, 33:02, Physician)

Some healthcare professionals demonstrated a strong **commitment to advocating for plwMS** in supporting the adoption of a brain‐healthy lifestyle. This included advocacy work to ensure the most appropriate and reliable services and resources are established to empower plwMS to completely embrace behaviour change:

And advocating for them to have those services. So writing their annual reports and things like that to make sure that they're getting, if they need nutrition supplements, what's the right one that they need? And making sure that they're getting funding for that. Do they need pre prepared meals through Lite N Easy or something like that? Do they need a support worker to help them with shopping and meal prep? Do they need support advocating for them? (P34, 8:52, Dietitian)

Although advocacy of brain health and a healthy lifestyle was perceived as a responsibility of healthcare professionals, such advocacy efforts were not consistently integrated between professional disciplines. Only a few incorporated these activities into their current practice, reflective of how that participant viewed/valued the importance of brain health. Nonetheless, healthcare professionals were seen to share an important responsibility, alongside the consumer, to **enable independence in practice and self‐care decision making**. Participants believed that plwMS should take ownership over their journey:

My role is to enable them to be independent, and they own their own journey. It's not my responsibility, as it were, I'm here to give advice and education, and I can give them as much information as I can. But then for them to take that. (P06, 36:55, Occupational therapist)

Independence was seen as important in self‐care decisions, which underpins the ability of the consumer to adopt and live a brain‐healthy lifestyle. Promoting independence in decision making and choice is an empowering concept for plwMS who can (and are assumed to) be actively and equally involved in self‐care decisions.

This is a bit of a roller coaster ride, and you've got no idea what's over the crest or around the corner. But it is your job to find and fasten your own seat belt, which is the shore knowledge of how to care for yourself. (P11, 34:45, Alternative therapist)

A majority of healthcare professionals were also able to recognise that behaviour change is a long‐term process, requiring **support and continuity of care** from a multidisciplinary MS care team:

It's also reinforcing that those lifestyle changes are a 40–50‐year journey, not ‘we'll get it all fixed in six months kind of thing … We're talking about decades of journey and how they put these little bits and pieces in place through that time period. (P20, 19:20, MS nurse)

Therefore, healthcare professionals were competent in acknowledging that long‐term behaviour change is not a quick‐fix solution, but rather, a journey that requires continual support, guidance and consistent messaging over the lifetime of their disease course:

So, if you tell a patient to stop smoking as a doctor, there's a 10% chance that they might then walk out and try to stop smoking, which isn't great. But if you know someone for their whole life, and you bring it up every single time, all those 10 precents add together. (P30, 22:27, Physician)

### 3.2 Theme 2: The collaborative role

The adoption of a brain‐healthy lifestyle by plwMS relies on a collaborative approach that **integrates** behaviour‐change strategies from a range of healthcare perspectives, expertise, and skill sets. Over time, the biomedical view of healthcare has evolved into a multidisciplinary team model, involving collaboration from at least two individuals from the following disciplines (presented in descending order according to the number of mentions in interview transcripts): neurologist, nurse specialist including MS nurse and continence nurse; physiotherapist, psychiatrist, psychologist, neuropsychologist, or counsellor; occupational therapist, exercise physiologist, dietitian or nutritionist; speech pathologist, personal trainer and/or naturopath.

The diversity of disciplines and roles within the MS care team reflected the complexities of MS and the multifaceted approach required to address the biopsychosocial, interpersonal, and contextual factors impacting brain health. Therefore, to integrate behaviour change strategies effectively, some healthcare professionals are observed to **delegate responsibility and coordinate care** from multiple sources of support:

So we have to prescribe what's tailored for that individual, something that they're able to do, and it's safe for them to do, and that they actually have the physical means to do it. And where help is needed, we also have to rely on help. So, if we need occupational therapy, or physiotherapy or exercise physiology, I think we need to, of course, engage our colleagues in that. (P09, 25:26, Physician)

The composition of the team varies and often reflects the consumers' medical and biopsychosocial needs as their disease ability changes over time:

It comes down to where they are in their disease process, what support they feel they need and what support I think they would benefit from. So, all these things need to come together. (P12, 8:54, Physiotherapist)

Most healthcare professionals demonstrated competence in defining their professional scope of practice and were seen to generate cross‐discipline referrals when required expertise, skills or experience exceeded their scope:

She went to see an OT [occupational therapist], because I've got to stay within my scope of practice … You know, you're making decisions which is somebody else's job, but you're not qualified to do it. So, point them in the right direction. (P02, 11:47, Exercise therapist)

The results revealed a significant challenge in an evident lack of a referral system. Cross‐disciplinary referrals were commonly ‘at the request of the participant [plwMS]’ (P36, 21:10, Dietitian) and tended to follow an ‘ad‐hoc’ approach. The challenges that exist in lacking a robust referral system were expressed with the most concern from healthcare professionals in the private sector:

I don't feel that there's a particular network going on… It is extremely difficult to navigate in a private practice environment because it's who's got the best website on Google ads. (P06, 20:49, Occupational therapist)

Alongside this, healthcare professionals also discussed the challenges in navigating between sectors of care, which are encumbered with systemic/environmental issues such as hierarchical structures between professional disciplines, time constraints and lack of role understanding. These factors were seen to heavily influence referral pathways and their initiation, particularly as most referral initiations are driven by primary physicians:

I don't necessarily initiate it. The initiation would come from the consult. But doctors just don't have time, or they just always forget how to do it. (P21, 8:15, MS nurse)

### 3.3 Theme 3: The communicative role

Healthcare professionals discussed the importance of **clear and concise communication strategies** to ensure that lifestyle and behaviour change information provided is understandable and can be practically adopted by plwMS. Additionally, participants acknowledged the importance of nonverbal communication as an important skill which was seen to influence the implementation of advice:

That's why it is so important to listen. You get such amazing ideas from people, and you sort of change the way you put things forward. (P22, 14:09, MS nurse)

Within this context, MS nurses and allied health professionals frequently acknowledged motivational interviewing to be an effective and ‘valuable tool’ (P03, 10:00, Psychologist) for understanding and uncovering motivations of plwMS to evoke and support change:

It's about asking the right questions to uncover the motivations of the person in front of you and help them get it done, but in a way that is meaningful for them. (P03, 9:27, Psychologist)

However, some allied health professionals, namely dietitians and physiotherapists, expressed concerns regarding a lack of training in effective communication and motivational interviewing:

I think more motivational interviewing support is needed for dietetics. Because all of us know our dietetics inside out. But it's being able to connect and make a change … it's more counselling. It's actually like we need a psychology degree as well. (P32, 37:16, Dietitian)

Psychologists, counsellors, and occupational therapists were identified by colleagues as most proficient in this skill, attributing their competence and effectiveness to their tertiary training:

Physio's and EPs [exercise physiologists] are really good at prescribing exercise. But we're not as good as other professions when it comes to motivating our clients, by using sort of the psychological principles of motivating them to change their behaviour. Whereas occupational therapists they have that underpinned in their undergraduate training more so than us. And then obviously psychologists and counsellors do too. (P10, 7:05, Physiotherapist)

Lastly, healthcare professionals described the critical **importance of consistent and constant messaging** between and within professional disciplines when recommending dietary and lifestyle management strategies for plwMS:

You have to have the constant message from the clinicians, but without the hampering. If you don't have the constant message that ‘this is just as important as the Tysabri [a prescription DMT used to treat MS, given by intravenous infusion], you know, you can't lay back on the banana lounge and just think that Tysabri is going to fix everything’. (P27, 36:21, MS nurse)

Results revealed that achieving this consistency is an ongoing and prominent challenge within the healthcare sector. Variations in consumer messaging were most prominent in the provision of dietary advice, with various and, in part, conflicting recommendations including:

We'd just be recommending a good balanced diet. That's what they recommend in the research from my understanding. (P04, 14:09, Occupational therapist)

We talk about intermittent fasting and not eating after nursing home hours really at six o'clock dinner and then maybe not until late morning the next day. (P11, 24:02, Alternative therapist)

The ones I advocate for are the Mediterranean or the MIND [Mediterranean‐DASH Intervention for Neurodegenerative Delay] diet. And you don't have to adhere to it 100%…. (P30, 3:55, Physician)

## 4 DISCUSSION

Recent research has been limited in pursuing the perspectives of how healthcare professionals perceive their role(s) in supporting behaviour change to optimise brain health among plwMS. To address this, we aimed to explore such perspectives of professional roles and current practices. Following one‐to‐one, semi‐structured interviews with Australian healthcare professionals with experience consulting plwMS, three themes were identified. The results were clear in that the impetus to behaviour change is a collaborative effort, driven largely by a shared responsibility between (A) plwMS themselves, who must take an active role to engage with and enact change, and (B) healthcare professionals. As research on brain health is evolving, it is evident that healthcare professionals may not yet completely appreciate its significance within the context of MS.

### 4.1 The empowering role

Participants indicated that their primary role was to empower plwMS by building awareness, understanding and preparedness for behaviour change to adopt a brain‐healthy lifestyle. Results uncovered a strong underlying assumption that was evident between disciplines. That is, plwMS will make appropriate lifestyle behaviour change(s) based on their level of prior knowledge. However, as we know, healthcare environments are laden with complexity and in some circumstances, there is an evident and unintentional disconnect between confirming consumer understanding and capability to enact change. Therefore, as reflected in our study, healthcare professionals appear to under‐estimate the complexity of converting knowledge into action,^26^ possibly exacerbated by a lack of understanding/awareness of the core concepts in the stages of behaviour change.^27^ These findings corroborate those of previous studies which have long showed that knowledge does not always translate into action,^28^ and further, that MS knowledge is not always associated with self‐efficacy.^29^ One qualitative study addressing physical activity behaviours in MS management reported that initiatives to increase engagement with health behaviours in populations such as MS, should also include behaviour change strategies to ensure optimal effectiveness of healthy lifestyle promotion.^30^ Strategies may include self‐efficacy assessment, barrier identification and problem solving.^31^ Hence, while education and empowerment are essential components to *promote* the adoption of a brain‐healthy lifestyle and *support* behaviour change, these components alone may not be sufficient to evoke change. An approach that seeks to bridge the gap between what is known and what is actually done, is needed.

Previous studies have attempted to explore the factors involved in this ‘knowledge to action gap’, such as personal characteristics, self‐efficacy, time constraints and physician perceptions,^32^ which are similar to those outlined in the SCT. As reflected in our study, additional consumer, clinician and organisational‐related barriers/forces are at play, including professional expertise and experience; tertiary education and training; consumer readiness to change, motivation(s) and health literacy, impeding how behaviour change takes action (Figure 1). These factors form important constructs of the SCT which have profound effect on determining human behaviour and outcomes.^33^ Consistent with the experiences from healthcare professionals from the United Kingdom's National Health Service, time constraints, organisational pressures, beliefs about capabilities and professional scope of practice were cited as a hinderance to healthcare professionals' ability to engage in conversations about lifestyle management and behaviour change.^34^ Similarly, qualitative work with physicians from Iran's primary healthcare system, as another example, identified similar personal factors of the healthcare professional and factors pertaining to the broader healthcare environment, which were found to influence consumer satisfaction, patient outcomes and the quality of medical services provided.^35^ Therefore, recognising these factors in the context of MS care is an important step in understanding system‐related issues and individual factors, and why/how these may influence translation.^36^ Further to this, Van de Ven^37^ argued that providers underestimate what is known about human behaviour, namely, that plwMS have poor attention skills and find remembering complex information challenging. Closing the gap between knowledge and action, therefore, requires an investment in high quality resources, training, skill sets and time by MS care providers, a strategy that has previously led to enhanced knowledge translation with actions to improve the quality of healthcare provided and received.^38^ These investments have been identified as effective in other healthcare systems.^35^

### 4.2 The collaborative role

When working collaboratively within a multidisciplinary team, an evident power differential and hierarchical structure between healthcare professionals was another key finding. While hierarchies cannot be abolished, this inevitable structure may negatively impact on behavioural change and how healthcare professionals work interdisciplinary. As reflected in our study, a case study of network and employment challenges in healthcare found similar interorganisational and interprofessional relationships that were a result of traditional hierarchies and power relations.^39^ Furthermore, misunderstanding of professional roles may inadvertently contribute to poor collaboration and conflict over who has the authority to make referral decisions, affecting trust and mistrust between consumers and healthcare providers, and interprofessionally.^40^

Our study builds on these findings and the results demonstrated that the onus is placed on plwMS to drive cross‐discipline referrals due to a lack of robust referral system. Hence, it was more common than not for consumers to take on the collaborative role of healthcare providers. This finding has been described in the literature for other chronic diseases^40^ and can be partially explained by the ideology of neoliberalism which places an emphasis on self‐responsibility, and the expectation that consumers will take proactive steps to maintain good health and a healthy lifestyle.^41^ Considering brain health is affected by a complex interplay of biological, psychological, social, interpersonal and contextual factors,^42^ individuals should not be held entirely responsible, which may contribute to the perceived difficulties in enacting change in this context.^43^ Interprofessional collaborative practice, as well as effective collaboration between healthcare professionals and plwMS, has been shown to facilitate consumer involvement in decision‐making, improved health outcomes, empowerment and security.^44^ Therefore, clarification of roles and role boundaries between and within disciplines and delineation of responsibilities and skill sets within each MS care team should be clearly defined as a means to encourage collaborative practice.^44^ This may foster a more supportive environment for plwMS seeking a brain‐healthy lifestyle.

### 4.3 The communicative role

Healthcare professionals considered motivational interviewing an important communication strategy that may support plwMS in their journey towards adopting a brain‐healthy lifestyle. However, concerns were raised regarding the lack of training in motivational interviewing skills. According to Seigart et al.,^45^ healthcare professionals who feel ill‐equipped to utilise motivational interviewing are less effective in their work compared to those with adequate training and field experience. Many motivational interviewing training courses for healthcare professionals exist,^46^ however greater recognition, with respect to implementation effects in MS care is needed. As discussed earlier, it is difficult to motivate if the underlying issues for change are yet to be addressed. Therefore, ongoing professional development and upskill in communication techniques across professional disciplines may enhance service delivery and further support behaviour change from effectively translating knowledge into action, across and between different healthcare settings for plwMS.

Lastly, the discordance in consumer healthcare messaging between disciplines is undoubtably an ongoing challenge for plwMS and may negatively contribute to the knowledge to action gap. Inconsistent healthcare information may adversely affect behaviour change and previous research has found that plwMS receiving conflicting advice from their healthcare professionals may lose trust and/or resort to less trustworthy information sources.^47^ This discordance is reflected in a recent scoping review summarising the current evidence on this topic.^42^ Participants acknowledged that there is an urgent need for healthcare professionals to be *singing from the same hymn sheet* (P24, 35:50, MS nurse) that plwMS can understand and translate into a brain‐healthy lifestyle. Knowledge translation strategies such as the development of guidelines, targeted toolkits containing printed education materials, tailored support, and training,^48^ may help clinicians to provide consistent and coordinated care, which may ultimately lead to improved consumer‐healthcare professional relationships and good decision making about MS self‐management.

### 4.4 Limitations and future research

There are limitations with qualitative research and efforts were taken to address these by the research team. First, the research quality is largely dependent on the moderators' skills and inherent biases, and as a result, the research teams' prominent position and training as dietitians may have influenced the data collected and the approach to analysis. Using analysts from other disciplines was conducted as was engagement with several reflexivity techniques, in attempt to minimise this bias. Second, underlying participant biases and idiosyncrasies may have also influenced their responses to our questions. Efforts were taken to ensure diverse sampling of participants from different professions, demographics, and contexts were included, and participants were reassured that there were no right or wrong answers to any questions.

Recruitment of accredited practising dietitian's with experience consulting plwMS was a noticeable challenge, most likely due to a lack of involvement in MS care and/or poor confidence in speaking about their experiences. This meant that the recruitment phase was extended to allow for an adequate sample to reach a diversity of participants. We also acknowledge that the particularities of the Australian healthcare context may limit transferability of the findings to healthcare systems and clinicians in other countries, and, therefore, results cannot be empirically generalised. That is, the findings of this research cannot be used to infer the characteristics to a wider population. Future research could examine the receptivity to behaviour change interventions by conducting longitudinal studies which aim to track effectiveness of different support strategies and their effects on optimising brain health for plwMS. The development and dissemination of MS guidelines to promote consistency in dietary and lifestyle recommendations may help to address the discordance in consumer healthcare messaging and support healthcare professionals to provide consistent messages.

Overall, the findings from this qualitative study hold promise for enhancing effective management planning that encompasses behavioural change support, co‐collaboration with other healthcare team members, and effective communication to facilitate improvements in brain health for plwMS. Greater awareness must be made in recognising and addressing the bidirectionality of external factors such as those in the SCT that may influence how behaviour change occurs. For example, the provision of personalised care plans, education, and counselling about the role of lifestyle factors in MS management and the incorporation of behavioural change strategies as central to routine healthcare discussions.

## 5 CONCLUSION

Participants in this qualitative study perceived their role in supporting the adoption of a brain‐healthy lifestyle to encompasses empowerment, collaboration, and communication both with plwMS, and, across disciplines. However, an unintentional disconnect is apparent where healthcare professionals under‐estimate the complexity of translating knowledge of behaviour change(s) into action. External factors/forces including time constraints, professional expertise, training (or lack of in some communication styles), skill set, consumer readiness, health literacy, self‐efficacy and motivation are at play, and can affect how behaviour change takes action. Future research should further explore how these factors, as well as professional hierarchies and discordant messaging, affect(s) knowledge uptake and behaviour change among plwMS, and may require further investment in training, skill sets and time by healthcare professionals. This is a crucial step in effective knowledge translation to improve the quality of healthcare provided and received in an MS setting.

## AUTHOR CONTRIBUTIONS

**Olivia Wills**: Conceptualisation; methodology; software; investigation; formal analysis; visualisation; project administration; writing—original draft; writing—review and editing. **Yasmine Probst**: Conceptualisation; methodology; investigation; supervision; visualisation; writing—review and editing. **Jodi Haartsen**: Visualisation; writing—review and editing; supervision. **Anne‐Therese McMahon**: Formal analysis; methodology; supervision; validation; visualisation; writing—review and editing.

## CONFLICT OF INTEREST STATEMENT

Olivia Wills receives a post graduate scholarship from Multiple Sclerosis Australia. Yasmine Probst has also received research grants from Multiple Sclerosis Research Australia, a fellowship from Multiple Sclerosis Australia, is a reviewer for a range of multiple sclerosis scientific journals, is a member of the Multiple Sclerosis Australia grant review panel and conference committee and has received honoraria from Multiple Sclerosis Research Australia and Multiple Sclerosis Plus. Yasmine is also a person living with MS. To minimise potential bias of personal connection to the research, Yasmine was distanced from the data analysis phase and had no direct contact with participants, interview recordings or transcripts. Jodi Haartsen is the Executive Management, Client Engagement and Wellbeing at Multiple Sclerosis Limited. Jodi was interviewed as a research participant before her invitation as a coauthor of this manuscript. Jodi was distanced from the data analysis phase and had no contact with participants, interview recordings or transcripts. Anne‐Therese McMahon declares no conflict of interest.

## ETHICS STATEMENT

Ethical approval for this study was obtained from the University of Wollongong Human Research Ethics Committee (reference number: 2022/334). Participants consented to the use of their data and deidentified exemplar quotes in publication.

## Supporting information

Supporting information.

Supporting information.

## Data Availability

The data that support the findings of this study are available from the corresponding author upon reasonable request.
