# Supplementary material for: The role of multidisciplinary MS care teams in supporting lifestyle behaviour changes to optimise brain health among people living with MS: A qualitative exploration of clinician perspectives
Source: Health Expect. 2024 Apr 4;27(2):e14042. doi: 10.1111/hex.14042 (PMC10995448; doi:10.1111/hex.14042)
Supplement: Supplementary file 2 — Supporting information. [file HEX-27-e14042-s002.docx]

**Supplementary material**

**Table S1.** Interview guide.

| **Question** |
| --- |
| 1. From your own perspective, what is your role as a [insert profession] working with people with MS? 2. Firstly, I want to understand what comes to mind when you hear or think of the concept “brain health?”  - *How would/do you associate brain health with MS?* - *What are your connotations or associations with “brain health?”* - *Is this something that you talk about in conversations with your clients with MS? How is it discussed?*  1. What comes to mind when you hear the phrase a “brain-healthy lifestyle?”  - *Does this mean the same to you as brain health?* - *Is this something that you talk about in conversations with your clients with MS? How so/how come?*  1. From your perspective, how might a [profession] support a brain-healthy lifestyle for people with MS?  - *Is healthy lifestyle management something that you have directly thought about before when speaking to plwMS?*  1. From your perspective, what are some ways that you encourage your clients to lead a brain-healthy lifestyle?  - *Tell me a little about why you support these factors?*  1. Previously diet was excluded from being classified under a brain-healthy lifestyle.  - *Do you feel diet has a role in the management of MS?* - *How is diet for plwMS discussed in a consultation?* - *How do you associate diet with MS?* - *Do you discuss dietary changes with people with MS as part of routine care? What dietary changes might you discuss?* - *What questions do plwMS ask about their diet? How do you respond?*  1. Now thinking about the team of clinicians that you work around, who do you think are the most critical team members that help support healthy lifestyle changes for people with MS?  - *What is their role in providing support to people with MS?* - *Who do you most frequently refer patients to and why?* - *Who most frequently refers patients to you?*  1. Thinking of a person with MS that you’ve consulted before that really embraced healthy lifestyle management, what were some key things that you think really **enabled** them to make positive changes? 2. Now thinking of another patient that wasn’t able to embrace healthy lifestyle management as much, what were some key things that you think acted as a **barrier** during the consultation for them to make positive changes?  - *How do you think some of these barriers could be addressed?* - *Do you feel you have received adequate training to confidently provide brain-healthy lifestyle advice to people with MS? (Eg. yes/ hindered, why?)*  1. Moving forward and thinking of ways that you, as a clinician, can further support the adoption of a brain-healthy lifestyle, what do you think might be helpful? 2. Is there anything else that you would like to bring up, discuss or do you have any more thoughts about this area?  - Is there anything else that comes to mind that you would like to discuss or share with me? - Please don’t hesitate to reach out via my email if you can think of anything else that you may have missed. |

**MS:** multiple sclerosis; **PlwMS:** people living with MS.
